# *Litopenaeus vannamei* Transcriptome Profile of Populations Evaluated for Growth Performance and Exposed to White Spot Syndrome Virus (WSSV)

**DOI:** 10.3389/fgene.2018.00120

**Published:** 2018-04-10

**Authors:** Camilla A. Santos, Sónia C. S. Andrade, Ana K. Teixeira, Flávio Farias, Karin Kurkjian, Ana C. Guerrelhas, João L. Rocha, Pedro M. Galetti, Patrícia D. Freitas

**Affiliations:** ^1^Departamento de Genética e Evolução, Universidade Federal de São Carlos, São Carlos, Brazil; ^2^Departamento de Genética e Biologia Evolutiva, Instituto de Biociências, Universidade de São Paulo, São Paulo, Brazil; ^3^Genearch Aquacultura, Rio do Fogo, Brazil; ^4^Aquatec Larvicultura de Camarão Marinho, Canguaretama, Brazil

**Keywords:** RNA-seq, growth, immune genes, WSSV, *Litopenaeus vannamei*

## Introduction

The Pacific white shrimp *Litopenaeus vannamei* represents one of the main marine shrimp species commercialized worldwide (Gucic et al., 2013; FAO, 2014; Senapati et al., 2017). Despite its importance on aquaculture and fishery activities, the exposure of wild and captive stocks to pathogens, such as fungi, bacteria and mainly virus, has commonly leading to concerns on healthy and population viability, causing damages related to quality of the shrimp used for local and international trading and human feeding (Wang and Wang, 2013; Liu et al., 2014; Rao et al., 2015). The White Spot Syndrome Virus (WSSV), for instance, may spread very quickly in the open sea, with even more severe harms for the aquaculture stocks, sometimes leading to 100% mortality (FAO, 2017). In addition to the animal health, growth is also an important value for the shrimp aquaculture (Liu et al., 2005; Jung et al., 2013; Lv et al., 2014). This scenario causes large economic losses on the global shrimp farming (Chen et al., 2015) and also on the annual fishing catches (Xue et al., 2013).

Despite the remarkable importance of penaeid shrimp, few data about determinant genes related to growth and immunity are already described for crustacean (Jindra et al., 2013; Jung et al., 2013; Lv et al., 2014), added to the fact that molecular mechanisms involved in growth and immunity are not yet known in detail. In this manner, it becomes evident the need of expanding the knowledge about genes and transcripts involved in cellular responses and physiology of this group. The lack of a shrimp reference genome highlights the importance of studies addressing next generation sequencing (NGS) as a relevant source of information (Guo et al., 2013; Santos et al., 2014; Powell et al., 2015).

In this study, we used the RNA-seq approach for assembling a robust *L. vannamei* transcriptome obtained from muscle tissue of captive individuals evaluated for growth and high survival performances, and also from hepatopancreas and muscle of animals exposed to White Spot Virus. Sequencing was carried out using Illumina technology (San Diego, California, USA), and the transcripts blastX annotation against Swissprot database returned more than 20 thousand unigenes with known gene products, identifying a higher amount than those previously reported for other marine shrimp transcriptomes (Nguyen et al., 2016; Rao et al., 2016). These findings certainly constitute an important source of genetic information for further studies, considering gene differential expression analysis and single nucleotide polymorphism (SNPs) identification in crustacean species.

## Value of the data

- *L. vannamei* has enormous commercial value among penaeid shrimp, which account for one-third of the annual global crustacean fisheries.
- The transcriptome reported herein was produced from hepatopancreas and muscle tissue samples of specific pathogen free (SPF) shrimp submitted to selective breeding programs for high rates of survival and fast growth; and to exposure to White Spot Virus.
- The percentage of unigenes presenting SwissProt hits described in this study is higher than those observed for other shrimp Illumina transcriptomes (e.g., Nguyen et al., 2016; Rao et al., 2016).
- These data certainly constitute an important source of genomic information for *L. vannamei* and other penaeid species in addition to provide resources for the aquaculture studies aiming higher growth gains and WSSV-resistant shrimp.

## Materials and methods

### Experimental design and sampling

The *L. vannamei* samples which were used in this work are SPF shrimp submitted to selective breeding programs for high rates of survival and fast growth in two shrimp commercial companies (Genearch and Aquatec), both located in Rio Grande do Norte (RN) state, Brazil. All samples were kept in a sanitary environment monitored for the presence of many pathogens, including WSSV and were collected in the first semester of 2015. The genetic improvement programs have been conducted based on performance of closed familiar lines, according to quantitative analyses and genetic gain parameters evaluated by the companies (data not available). For the sampling of individuals evaluated for growth and survival performance, 20 closed families in triplicates were assessed, and four families with the higher and four with the lower growth rates were selected. We sampled muscle tissue (pleopods) of one animal from each family, totalizing eight animals evaluated for growth performance. The individuals were about 2 months aged and weighed around 1.5 g. For the WSSV exposed shrimp sampling, we used SPF individuals of a hybrid line from Aquatec, which was originated from the crossing of two different SPF lines, one from Aquatec and another from Genearch. These hybrid shrimps were exposed to WSSV in a farm pound with a high incidence of the disease. Before and after the exposure to the virus, shrimp hemolymph samples were submitted to quantitative real-time PCR (qPCR) analyzes in order to confirm the presence or absence of the WSSV, following a procedure proposed by Silva et al. (2011). The efficiency of qPCR amplification was determined by the method described by Pfaffl et al. (2002) and the specificity of the amplification was confirmed by the analysis of the dissociation curve (Ririe et al., 1997). After the contact with the WSSV and qPCR tests, we selected one positive animal showing WSSV symptoms, and a negative one with no symptoms, both weighting about 6 g. Later, hepatopancreas and muscle tissues from each individual were sampled, totalizing four samples from these two shrimps evaluated to WSSV. Hepatopancreas and muscle tissues were chosen given their relevance to immune response in crustacean (Fan et al., 2016), and as a target for pathogen infections, including WSSV (Yu et al., 2017), respectively. The tissue samples were conditioned in RNA later (Thermo Fisher Scientific, Waltham, MA, USA) and kept in biofreezer (−80°C) for the isolation of total RNA.

### RNA isolation and cDNA library preparation

In total, 12 cDNA libraries were established. Eight libraries were constructed using muscle tissue of individuals evaluated for growth performance; the four remaining ones were constructed using muscle and also hepatopancreas from the two individuals exposed and evaluated to WSSV by qPCR. Total RNA isolation was performed using the Trizol®/chloroform protocol (Chomczynski and Mackey, 1995), modified for the centrifugation time in ethanol washing (12 min) and added a purification stage using lithium chloride (7.5 M) for elimination of phenol traces. The quality was assessed in a Qμbit fluorometer (Thermo Fisher Scientific), quantity was evaluated in a NanoDrop spectrophotometer (Thermo Fisher Scientific) and samples with ratios between 1.8 and 2.2 were considered pure. The integrity of the samples was confirmed in a BioAnalyser equipment (Agilent Technologies Inc., Santa Clara, CA, USA) and those with RNA Integrity Number (RIN) ≥ 6 were stored at −80°C. A cDNA library was produced from each sample using a TruSeq RNA Library Preparation V2 kit (Illumina Inc., San Diego, California, USA).

### Sequencing, *de Novo* assembly and BlastX annotation

Sample quantification was carried out in qPCR with the Universal Library Quantification Kit (KAPA Biosystems, Wilmington, MA, USA), prior to loading into the sequencer. The libraries were grouped and ran on the Illumina HiSeq 2500 platform with 2 × 100 bp paired-end, using a TruSeq SBS V3 kit (Illumina Inc., Thermo Fisher Scientific), at the Laboratório de Biotecnologia Animal, Escola Superior de Agricultura “Luiz de Queiroz”—Universidade de São Paulo (ESALQ-USP). The quality of the raw data generated after sequencing was visualized in the FastQC software (version 0.10.1) (http://www.bioinformatics.babraham.ac.uk/projects/fastqc/). All reads were filtered for Phred quality (QS) 23 (sequence inside) and 30 (sequence edges) and minimum length of 65 bp using the SeqyClean software (v.1.9.9) (https://github.com/ibest/seqyclean). The same package was also used to remove contaminant sequences (primers and vectors) using the Univec database (https://www.ncbi.nlm.nih.gov/tools/vecscreen/univec/). After filtering, the reference *de novo* assembly was performed in the Trinity software (Grabherr et al., 2011). The sequences were normalized using Trinity's insilico_read_normalization.pl script, with 50X adopted as the minimum coverage value. For the assembly, 300 bp was defined as the minimum size of the assembled contigs. The CD-Hit package (Li and Godzik, 2006) and DustMasker (Morgulis et al., 2006) were used to remove redundant contigs with more than 95% similarity and low complexity sequences, respectively. The TransDecoder package (http://transdecoder.sourceforge.net/) was used to identify the contigs candidate coding regions. Meanwhile, Trinotate pipeline (https://trinotate.github.io/) was employed for annotation of the sequences through the following databases: Uniprot (uniref90 + SwissProt) with cut-off value of 1e10^−5^, Gene Ontology (GO) (Ashburner et al., 2000) for the GO terms Biological Process, Molecular Function and Cellular Component and KEGG (Kyoto Encyclopedia of Genes and Genomes) (Kanehisa et al., 2012), with the identification of the main metabolic pathways found. The paired reads fastq files from all the samples used in transcriptome analysis (BioProject: *Litopenaeus vannamei* hepatopancreas and muscle Transcriptome) are available in the NCBI Sequence Read Archive (SRA) under the accession number SRP128934. This Transcriptome Shotgun Assembly project has been deposited at DDBJ/EMBL/GenBank under the accession GGKO00000000. The version described in this paper is the first version, GGKO01000000.

## Results and discussion

The 488,681,310 transcripts from the 12 libraries were filtered on SeqyClean for contaminants, rRNA and low quality sequences. From these, about 30 million paired reads (29,376,967) remained after normalization in Trinity package, resulting in 28,654,475 (97.5%) fragments used in the reference transcriptome after filtering. We obtained 63,105 transcripts with 2,511 bp mean length and N50 of 3,464 bp. From this total, 20,865 (33%) showed blastX hits on SwissProt database (Supplementary Material 1; Table 1). The contigs filtered to <95% of similarity were considered unigenes after the reference assembly. The contig average size found here was greater than those previously reported in *L. vannamei* (Ghaffari et al., 2014), *Penaeus monodon* (Nguyen et al., 2016) and *Macrobrachium rosenberguii* (Rao et al., 2015, 2016) Illumina transcriptomes. The longest contig size observed in our study was equal to 20,061 bp, being superior to the value of 13,578 bp reported for *P. monodon* transcriptome (Nguyen et al., 2016). After elimination of the redundant contigs, low complexity sequences and isoforms, candidate-coding regions were identified, remaining 14,124 unigenes. The *de novo* assembly and the automatic annotation results are shown in Table 1.

**Table 1:** Statistics of the reference assembly after normalization and an overview of the functional annotation of transcripts.

**Transcripts used in the construction of the reference after filtering on SeqyClean**						
	**Growth**		**WSSV-exposure**			
**Tissue**	**Muscle (8 libraries)**		**Muscle (2 libraries)**		**Hepatopancreas (2 libraries)**	
**Condition**	**Higher growth**	**Lower growth**	**WSSV-negative**	**WSSV-positive**	**WSSV-negative**	**WSSV-positive**
Paired-end fragments	142,094,916	150,593,354	44,029,454	44,972,016	59,850,408	47,141,162
Total	292,688,270		89,001,470		106,991,570	
			195,993,040			
488,681,310						
**Transcripts after normalization in Trinity**						
Reads number after normalization					29,376,967	
Remaining reads number after low quality and adaptors filtering/ Reads number used in reference *de novo* assembly					28,654,475 (97.5%)^*^	
**Reference transcriptome analysis**						
Total number of contigs produced					63,105	
SwissProt blastX hits unigenes					20,865 (33%)^**^	
Average size					2,511 bp	
N50					3,464 bp	
Longest transcript (pb)					20,601	
Transcripts number > 1,000 bp					27,887	
Number of non-redundant unigenes with ORFs					14,124	

(*) from total reads number after normalization and

(**) *from total number of contigs produced*.

**Figure 1:**
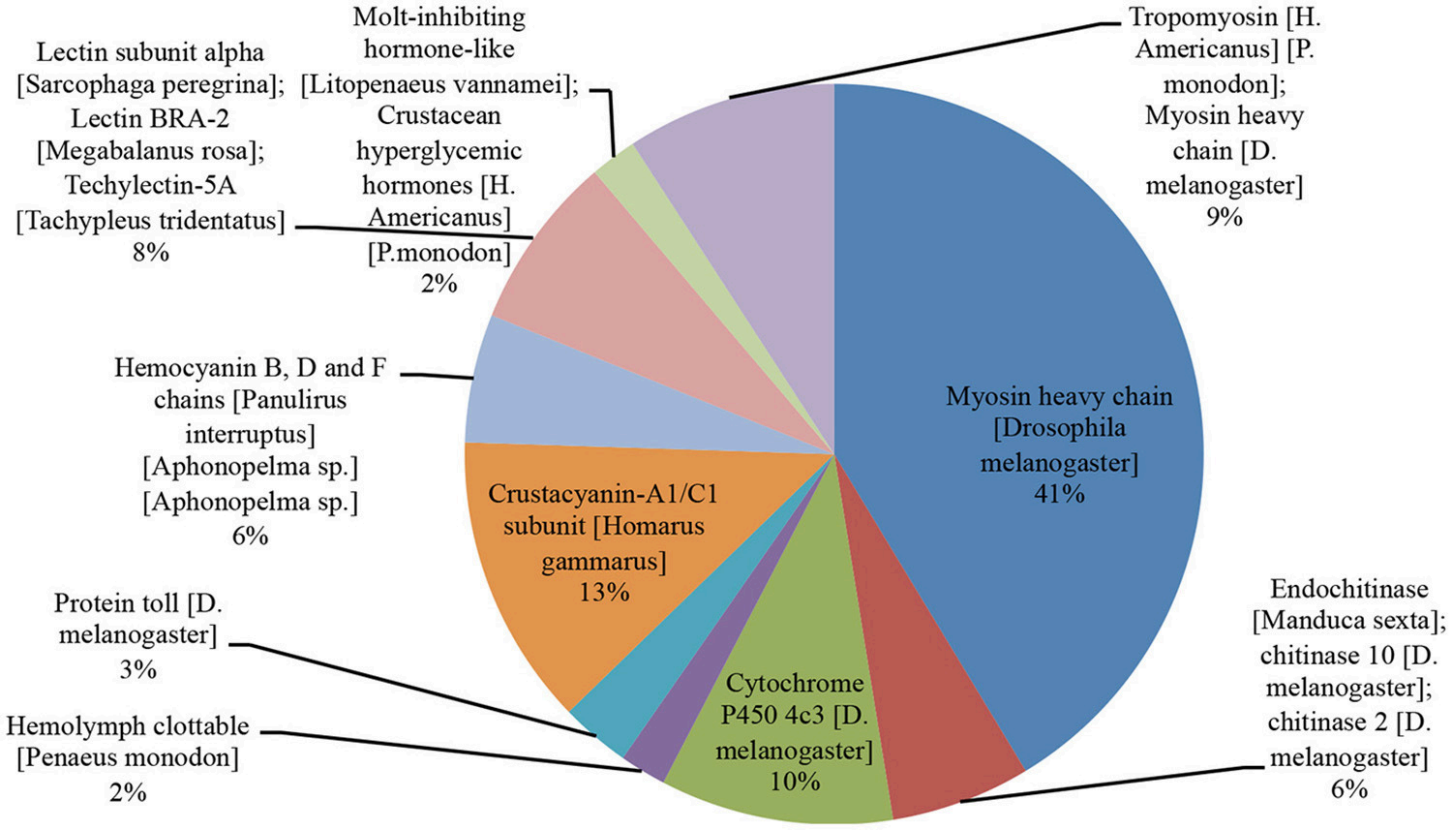
SwissProt blastX hits found for the top 10 proteins related to growth and immunity in arthropod species. The protein name and blastX hit in arthropod species are shown. Most of the blastX returned hits for myosin heavy chain (constituent of muscle) (41%) and crustacyanin (13%) (acting against virus in crustacean) proteins.

Following the sequences alignment against SwissProt database, the focus was given to genes that showed hits for arthropod species and fitness-related functions, such as growth and disease resistance. Among the most frequent proteins exclusively identified in the animals evaluated for growth performance are those related to myosin (MYSA) (41%), tropomyosin and myosin heavy chain (9%), chitinase (6%) and molt-inhibiting hormone-like (2%) genes (Figure 1). Myosin acts on the formation of the muscular myofibrils and, along with the paramyosin, is responsible for the stabilization of the sarcomere (Liu et al., 2005). There is evidence that increased myosin production and reorganization of myofibrils is associated with growth during and after shrimp molt (Cesar and Yang, 2007; Li et al., 2014). Chitin constituent proteins are among those responsible for the digestion of arthropod chitin exoskeleton, enabling molt and animal growth (Li et al., 2015). Among the most frequent genes in the WSSV-exposed shrimp samples returning blastX hits were mainly those related to crustacyanin 1 (CRA1) and 2 (CRA2) (13%), lectins (8%) and hemocyanin B (HCYB), and C (HCYC) heavy chain (6%) (Figure 1). Hemocyanin and crustacyanin proteins have a wide range of action against viruses, especially WSSV, acting on animal stress and survival responses (Fan et al., 2016), agglutination of the pathogen on hemolymph and cell lysis (Shockey et al., 2009; Zheng et al., 2016). On the other hand, lectins act on the pathogen specific recognition through molecular structures to a given molecule (Song et al., 2010).

The *de novo* transcriptome assembly from muscle and hepatopancreas of non-infected and infected shrimp, enabled the identification of important genes with potential fitness-related functions involved in growth and immunity. Such assembly strategy, gathering two different tissues with diverse functions, was performed in the effort to reach a wider range and representativeness of transcripts, producing the most robust transcriptome dataset for *L. vannamei* reported to date. It becomes of particular interest for the species lacking a reference genome, such as the majority of crustacean, including *L. vannamei*. Considering the high accuracy and wide coverage of RNA-seq produced here, this dataset can be useful for a gamma of further approaches, such as differential gene expression studies, analyses of isoforms originated from alternative splicing (Rao et al., 2015; Sun et al., 2015), SNPs calling (Cui et al., 2014; Santos et al., 2014; Yu et al., 2014), with potential to high-density chip and linkage map (Baranski et al., 2014), apart from genome wide association (GWAS) studies (Yu et al., 2017).

## Author contributions

CS has contributed to the transcriptome experiments, data analysis, and article writing and discussion; SA has conducted the bioinformatics analyses and participated in the article writing and discussion; KK has performed the qPCR analyses and the experiments related to the WSSV exposure; JR, FF, AT, and AG have conducted the selective breeding programs in the Genearch and Aquatec companies; PG has collaborated in the article writing and discussion; PF has lead the work, participating in the data analyses, article writing and discussion.

### Conflict of interest statement

The authors declare that the research was conducted in the absence of any commercial or financial relationships that could be construed as a potential conflict of interest.

## Supplementary material

Supplementary Material 1 All the *blastX* hits found for 20,865 transcripts in the SwissProt database obtained for *Litopenaeus vannamei*.

## Abbreviations

PLs: post-larvae
SPF: Specific Pathogen Free
WSSV: White Spot Syndrome Virus.
